# Paleogenetic study on the 17^th^ century Korean mummy with atherosclerotic cardiovascular disease

**DOI:** 10.1371/journal.pone.0183098

**Published:** 2017-08-16

**Authors:** Dong Hoon Shin, Chang Seok Oh, Jong Ha Hong, Yusu Kim, Soong Deok Lee, Eunju Lee

**Affiliations:** 1 Bioanthropology and Paleopathology Lab, Department of Anatomy, Seoul National University College of Medicine, Seoul, South Korea; 2 Institute of Forensic Science, Seoul National University College of Medicine, Seoul, South Korea; 3 Department of Forensic Medicine, Seoul National University College of Medicine, Seoul, South Korea; 4 Department of Internal Medicine, Asan Medical Center, University of Ulsan College of Medicine, Seoul, South Korea; University of Florence, ITALY

## Abstract

While atherosclerotic cardiovascular disease (ASCVD) is known to be common among modern people exposed to various risk factors, recent paleopathological studies have shown that it affected ancient populations much more frequently than expected. In 2010, we investigated a 17^th^ century Korean female mummy with presumptive ASCVD signs. Although the resulting report was a rare and invaluable conjecture on the disease status of an ancient East Asian population, the diagnosis had been based only on anatomical and radiological techniques, and so could not confirm the existence of ASCVD in the mummy. In the present study, we thus performed a paleogenetic analysis to supplement the previous conventional diagnosis of ASCVD. In aDNA extracted from the same Korean mummy, we identified the risk alleles of seven different SNPs (rs5351, rs10757274, rs2383206, rs2383207, rs10757278, rs4380028 and rs1333049) that had already been revealed to be the major risk loci of ASCVD in East Asian populations. The reliability of this study could be enhanced by cross-validation using two different analyses: Sanger and SNaPshot techniques. We were able to establish that the 17^th^ century Korean female had a strong genetic predisposition to increased risk of ASCVD. The current paleogenetic diagnosis, the first of its kind outside Europe, re-confirms its utility as an adjunct modality for confirmatory diagnosis of ancient ASCVD.

## Introduction

Analyses on human remains from archaeological sites are very useful for obtaining knowledge on the health and disease statuses of our ancestors [1–3]. Especially in the case of mummies, as their preservation status is far better than those of other types of archaeological corpses, paleopathological data attained from them tend to be much more valuable [4–6]. In addition to conventional diagnostic tools such as anatomical or radiological techniques, recently developed genetic analysis has deepened our understanding of past diseases from another perspective [7–9].

In South Korea, there have been a number of reports on mummies recovered from 15^th^-to-18^th^ century Joseon tombs. During the past 10 years, those scientific studies have provided us with significant data for comprehensive understanding of Joseon peoples’ health and disease statuses, which knowledge could not easily be attained by historical and archaeological investigations [6,10–23]. Particularly by ancient DNA (aDNA) analysis on Korean mummy samples [24–32], the genetic backgrounds of specific diseases prevailing in the past could be traced.

In 2010, during a computed tomography (CT) examination, we discovered multiple aortic calcifications in a 17^th^ century Korean female mummy (nicknamed *Mungyeong*). We suspected that the calcifications represented atherosclerosis of the aortic wall. This radiological finding was confirmed by subsequent autopsy in which multiple signs of aortic atherosclerosis were found once again. The mummified female must have suffered from coronary artery disease (CAD), as intimal thickening was also discovered in the left anterior descending (LAD) artery. Actually, this is a rare report of remnant atherosclerotic cardiovascular disease (ASCVD) in ancient human remains of East Asia [18].

Despite its academic implications, however, we also admit that the previous report was not sufficient to capture the full aspects of ancient ASCVD. Actually, a genetic predisposition to cardiovascular disease had already been proven in the paleogenetic study on “the Iceman,” a 5,300-year-old chalcolithic mummy [33,34]. This study showed, for the first time, that genetic analysis can be very successful in diagnosing ancient atherosclerosis. However, as this is only report of its kind to date, it is difficult to be sure that the same technique could also be applied to the diagnosis of similar cases discovered in any countries or continents.

The purpose of this study therefore was to determine whether paleogenetic analysis can also be useful for cases such as that of the above-noted 17^th^ century Korean mummy. More specifically, we attempted to determine if paleogenetic investigation by analysis of single-nucleotide polymorphism (SNP) can be an adjunct modality to anatomical or radiological analyses for confirmatory diagnosis of ASCVD in ancient human remains.

## Materials and methods

### Archeological information

**Fig 1:**
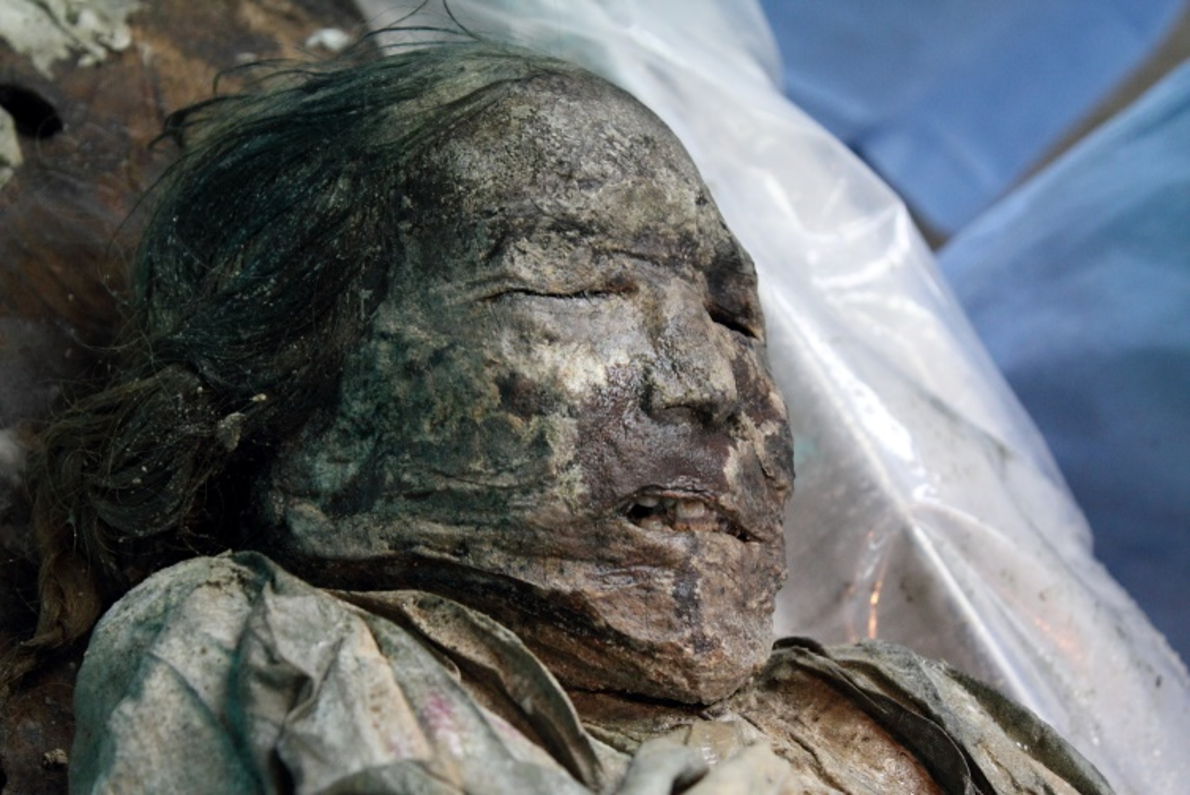
*Mungyeong* mummy examined in this study.

In April 2010, a middle-aged female mummy (repository number #278, JDHS Collection of Seoul National University College of Medicine, Seoul, South Korea) was discovered within a Joseon tomb in *Mungyeong* County, South Korea (Fig 1). The approximate time of burial, as estimated by a tree-ring test of the coffin wood, was the 1560s CE. The anatomical and radiological findings observed in this case are summarized in our previous report [18].

In brief, aortic calcifications were found on CT images. These findings were confirmed to be atheromatous plaques on autopsy. We also found other signs suggestive of atherosclerosis, such as ulcerated plaques, ruptured hemorrhages, and intimal thickening in which the necrotic core was covered by the fibrous cap. In the LAD, we also discovered intimal thickening and protrusion into the lumen, a definite sign of CAD [18]. We strongly suspected that the *Mungyeong* mummy had suffered from ASCVD.

### aDNA extraction

This study was declared by the Institutional Review Board (IRB) of Seoul National University Hospital as an exempt (IRB no. 2013–004). It was conducted in accordance with the Vermillion Accord on Human Remains, World Archaeological Congress (South Dakota, 1989).

The brain tissue of the *Mungyeong* mummy was subjected to aDNA analysis. The sample (0.5–1 g) was incubated in 4 mL of lysis buffer (EDTA 50 mM, pH 8.0; 1 mg/mL of proteinase K; SDS 1%; 0.1M DTT) at 56°C for 24 hr. Total DNA was extracted with an equal volume of phenol/chloroform/isoamyl alcohol (25:24:1) and then treated with chloroform/isoamyl alcohol (24:1). DNA isolation and purification was performed using a QIAquick PCR purification kit (QIAGEN, Hilden, Germany). The purified DNA was eluted in 50 μl of EB buffer (QIAGEN) [26,27].

### Polymerase Chain Reaction (PCR), cloning and sequencing

We selected 10 SNPs (rs5351, rs10757274, rs2383206, rs2383207, rs10757278, rs6903956, rs4380028, rs10953541, rs974819 and rs1333049) linked with atherosclerosis, CAD or myocardial infarction (MI) reported for European, South Asian, Chinese, Japanese and South Korean populations [33,35–43]. The relevant information on the selected SNPs is summarized in Table 1.

**Table 1:** PCR primer sets and SNP analysis results by Sanger sequencing.

Related Disease	dbSNP	Primer sequence (5' → 3')		Product size (bp)	AT(℃)	SNP	Risk allele	Allele	Primer reference
Atherosclerosis	rs5351^1^	Forward	TCA TCC CTA TAG TTT TAC AAG ACA GC	74	58	A/G	A	10A	Keller et al., 2014
		Reverse	ATG GCC AAT GGC AAG CAG A						
CAD/CA/CHD	rs10757274^2^^,^^3^^,^^4^	Forward	CCC CCG TGG GTC AAA TCT AAG	82	58	A/G	G	9G,1A	Keller et al., 2014
		Reverse	AGA ATT CCC TAC CCC TAT CTC CTA TCT						
CAD/CA	rs2383206^3^^,^^4^	Forward	TAC TAT CCT GGT TGC CCC TTC TGT C	78	58	A/G	G	9G	Keller et al., 2014
		Reverse	GGT TCA GGA TTC AGG CCA TCT TG						
CA/MI	rs2383207^4^	Forward	GAT ACT TAG CCC TTG GGA CC	121	58	A/G	G	10G	this study
		Reverse	TTG GGC AGC TCT TTT CAT AC						
CA/MI	rs10757278^4^	Forward	GAC AGG GCT GTG GGA CAA GTC	83	58	A/G	G	10G	this study
		Reverse	AGA GAA GGA GAA ACT ACT CTG TC						
CAD/CA/MI	rs6903956^5^^,^^6^	Forward	GGG GAA CAG AGA GAG ATT CCA TC	127	58	A/G	A	8G	this study
		Reverse	GCA CCC ACT TCA ACA CTT GG						
CAD	rs4380028^7^	Forward	ACT GGG GCA TGG AAA GGT TA	116	58	C/T	C	9C	this study
		Reverse	AGA GGC AGT GAT ATG GAG CG						
CAD	rs10953541^7^	Forward	GTG TGC CTC TTG AGG ATA AAG C	150	58	C/T	C	9T	this study
		Reverse	AGG GTT CTG TTT CCT GGT CC						
CAD	rs974819^8^^,^^9^	Forward	ACA CCA TGG ACA AAG AGA AAA	163	53	C/T	T	10C	this study
		Reverse	TGT ATG TAT AAG CAG GGG ATA ACT						
CAD/CA/CAC	rs1333049^9^^,^^10^	Forward	CTG CTT CAT ATT CCA ACT TGT GT	139	56	C/G	C	8C	this study
		Reverse	TTG CTT ACC TCT GCG AGT G						

CAD, coronary artery disease; CA, Coronary atherosclerosis; CHD, coronary heart disease; MI, Myocardial infarction; AT, annealing temperature. SNP, single nucleotide polymorphism; AT, annealing temperature. SNP references

^1^Yasuda et al., 2007

^2^Xie et al. 2011

^3^Keller et al., 2012

^4^Shen et al., 2008

^5^Wang et al., 2011

^6^Guo et al. 2012

^7^C4D genetics consortium, 2011

^8^Zhou et al., 2012

^9^Dechamethakun et al., 2014

^10^Hinohara et al., 2008.

Ten μl of aDNA extracted from the brain-tissue sample was treated with 1.0 unit of uracil-DNA-glycosylase (New England Biolabs, MA, USA) for 30 min at 37°C. Then, aDNA (40 ng) was amplified by PCR in a 20 μl reaction mixture containing a 200 μM dNTP mixture (iNtRON Biotechnology, Seoul, Korea), 1 mg/ml of BSA (New England Biolabs, MA, USA), 10 pmol of each primer (Integrated DNA Technologies, IA, USA), 1X GeneAmp® gold buffer, 2 mM MgCl_2_, and 2 units of Amplitaq gold DNA polymerase (Applied Biosystems, CA, USA).

The PCR conditions were as follows: pre-denaturation at 95°C for 10 min, 50 cycles of denaturation at 95°C for 30 sec, annealing at 53–58°C for 30 sec, extension at 72°C for 30 sec, and final extension at 72°C for 10 min. The PCR products were separated on 2.5% agarose gel (Invitrogen, Carlsbad, CA, USA) and then stained with ethidium bromide. Also, negative controls (extraction controls) were applied to gel electrophoresis. We photographed the results with a Vilber Lourmat ETX-20M equipped with Biocapt software (Vilber Lourmat, Collégien, France).

Cloning and sequencing were performed for amplified PCR products of the expected sizes. After the DNA of the amplified bands was extracted using the QIAquick Gel Extraction Kit (QIAGEN, Hilden, Germany), bacterial transformation was completed using the pGEM-T Easy Vector system (Promega, WI, USA). Bacteria transformed by the DNA products were grown on an agar plate containing ampicillin (100ug/ml), 0.5 mM IPTG and X-GAL (40 ug/μl) for 12 -14hr.

The selected colonies were grown in LB media for 12–16 hr, after which plasmid was harvested using the QIAprep spin miniprep kit (QIAGEN, Hilden, Germany). Sequencing was performed on each strand using the ABI Prism BigDye Terminator v3.1 Cycle Sequencing Ready Reaction Kit (Applied Biosystems, CA, USA) and the 3730xl Automated Sequencer (Applied Biosystems, CA, USA). The consensus sequences were determined using Alignment Explorer implemented in MEGA7 [44]. The SNPs obtained from Sanger sequencing were observed and compared with the risk alleles of each locus.

### SNaPshot analysis

We used 10 single-base-extension (SBE) primers for SNP analysis (Table 2) with each of the PCR products (1–3μl). The SBE reactions were carried out using a SNaPshot™ Kit (Applied Biosystems, CA, USA) and a PTC-200 DNA Engine (Bio-Rad Laboratories, CA, USA), following the manufacturer’s instructions. The thermal cycling conditions were as follows: 25 cycles of denaturation at 96°C for 10 sec; annealing at 50°C for 5 sec; extension at 60°C for 30 sec. The amplicons were purified with 1.0 unit of shrimp alkaline phosphatase (SAP) (USB Corporation, OH, USA), incubated at 37°C for 45 min, and heat-inactivated at 80°C for 15 min. The reactants were analyzed by an ABI PRISM 3100 Genetic Analyzer (Applied Biosystems, CA, USA) and GeneMapper^®^ID software, v3.2.1 (Applied Biosystems, CA, USA). The SNP scoring at each locus was confirmed by repeated SNaPshot analysis. The Sanger sequencing and SNaPshot experiments were conducted independently in different laboratories, and the results were compared.

**Table 2:** SNP analysis of *Mungyeong* mummy DNA by SNaPshot Kit.

**dbSNP**	**SBE Primer sequence (5' → 3')**	**SNP**	**Risk allele**	**Allele (PCR1)**	**Allele (PCR2)**
rs5351	TAC AAG ACA GCA AAA GAT TGG TGG CT	A/G	A	A	A
*rs10757274	TCT ATC TAG TGA ATT TCA ATT ATG TC	A/G	G	G	G
*rs2383206	TTC AGG ATT CAG GCC ATC TTG CAA A	A/G	G	G	G
rs2383207	TTT TTT ACT CCT GTT CGG ATC CCT TC	A/G	G	G	G
*rs10757278	CTG TCT TGA TTC TGC ATC GCT GC	A/G	G	G	G
*rs6903956	TTG GGG GAC CAA CCT TAA GTA ATA A	A/G	A	G	G
rs4380028	GGC ATG GAA AGG TTA AGT AAC TTG	C/T	C	C	C
rs10953541	TAT GGG TAC CTA AGT ATT AGC AGC A	C/T	C	T	T
*rs974819	TCT CCA AAC ATG AAA ATA AAA CAG TA	C/T	T	C	C
rs1333049	CAT ACT AAT CAT ATG ATC AAC AGT T	C/G	C	C	C

*Reverse direction

### Authenticity of this study

In the course of the sampling and lab work, we wore sterilized protection gloves, masks, gowns, and head caps. Our aDNA lab facilities had been set up according to the protocol of Hofreiter et al. [45]. The rooms for aDNA extraction and PCR preparation, respectively, were physically separated from our main PCR lab. The DNA extraction/PCR preparation rooms were each equipped with UV irradiation, isolated ventilation, and a laminated flow hood. Every procedure in this study was performed according to the authentic aDNA analysis criteria suggested by Hofreiter et al. [45].

We also tried to see whether the modern DNA might contaminate the ancient samples used in this study. The mtDNA profiles of all of the researchers involved were determined with the permission of the Institutional Review Board of Seoul National University (H-0909-049-295).

After obtainment of the mtDNA control region sequences from the mummy and researcher samples [46–48], 40 ng of aDNA was mixed with premix solution containing 1X Ampli*Taq* Gold® 360 Master Mix (Life Technologies, CA, USA) and 10 pmol of each primer (Integrated DNA Technology, IA, USA). The current PCR conditions and primer sequences were the same as in our previous study [28, 49]. The PCR products, as isolated using the Qiagen gel extraction kit (QIAGEN, Hilden, Germany), were sequenced by the ABI Prism® 3100 Genetic Analyzer (Applied Biosystems, CA, USA) using the ABI Prism® BigDye™ Terminator Cycle Sequencing Ready Reaction Kit (Applied Biosystems, CA, USA). The obtained sequences were aligned using Alignment Explorer implemented in MEGA7 [44] for determination of the mummy’s and researchers’ mtDNA consensus sequences, which subsequently were compared to rule out any possibility of contamination.

## Results

**Fig 2:**
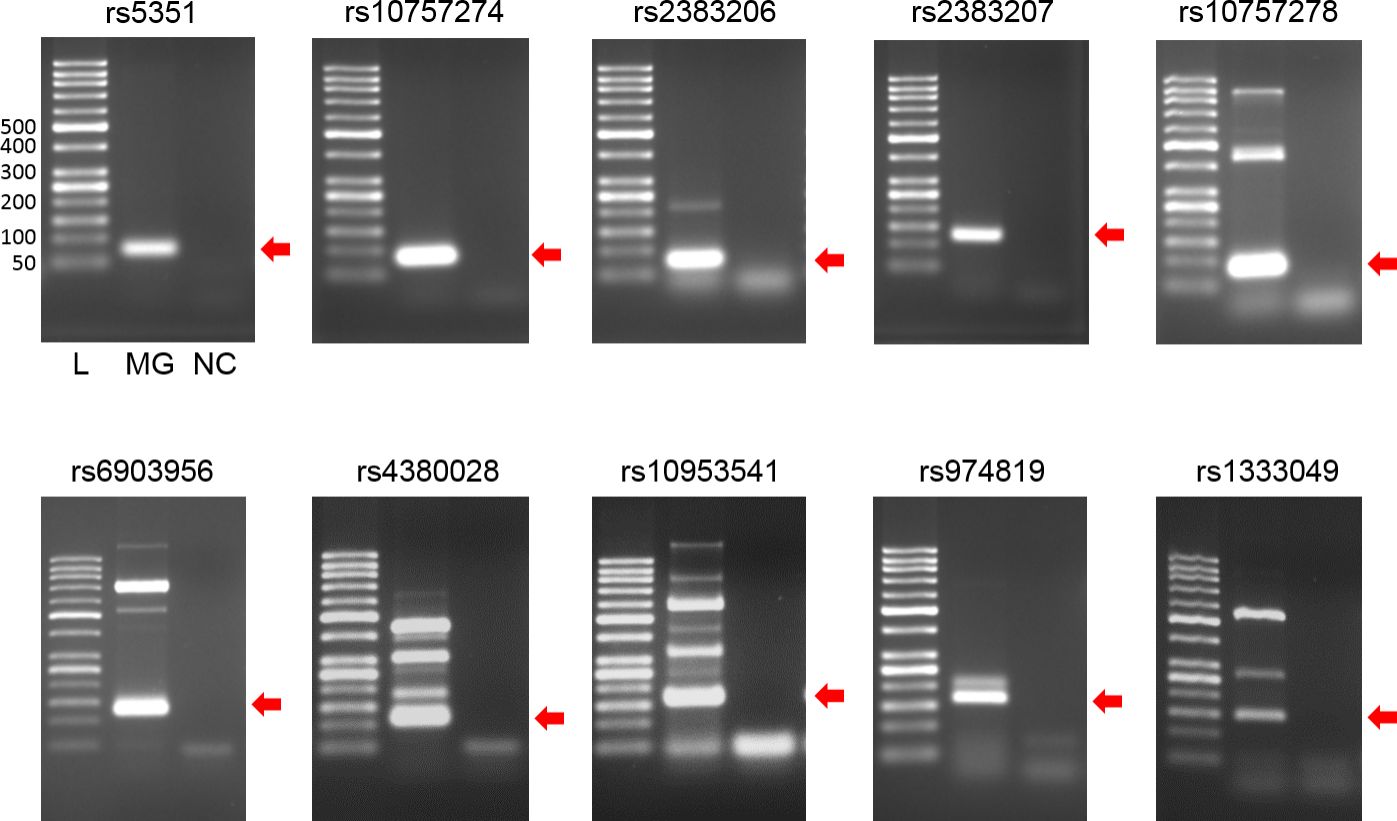
PCR amplification of DNA extracted from *Mungyeong* mummy sample. Amplicons associated with each SNP site were observed (indicated by arrows). L, 50bp ladder; MG, *Mungyeong* mummy; NC, negative extraction control.

In the gel electrophoresis of the PCR amplicons for the 10 different SNPs, we found amplified bands of the expected sizes at each target locus. Meanwhile, there were no bands detectable for the negative control samples (Fig 2). For the amplified DNA, cloning and sequencing analyses were then performed. From each DNA amplicon of the target loci, between 8 and 10 clone sequences were obtained (S1 File). By multiple-sequence alignment using the ClustalW program implemented in MEGA7 [44], we genotyped each target SNP.

The SNP genotypes obtained from the consensus sequencing results were screened to determine the presence of risk alleles. Among the SNPs examined, the risk alleles of atherosclerosis were found in seven different SNPs (rs5351, rs10757274, rs2383206, rs2383207, rs10757278, rs4380028 and rs1333049). Specifically, homozygous genotypes were identified at rs5351 (AA), rs2383206 (GG), rs2383207 (GG), rs10757278 (GG), rs4380028 (CC), and rs1333049 (CC) (Table 1). In the case of rs10757274, the SNP genotype determined by Sanger sequencing was 9G, 1A. No risk alleles were found in three SNPs (rs6903956, rs974819, rs10953541) even though they also had been established as atherosclerosis-related loci for CAD and MI.

**Fig 3:**
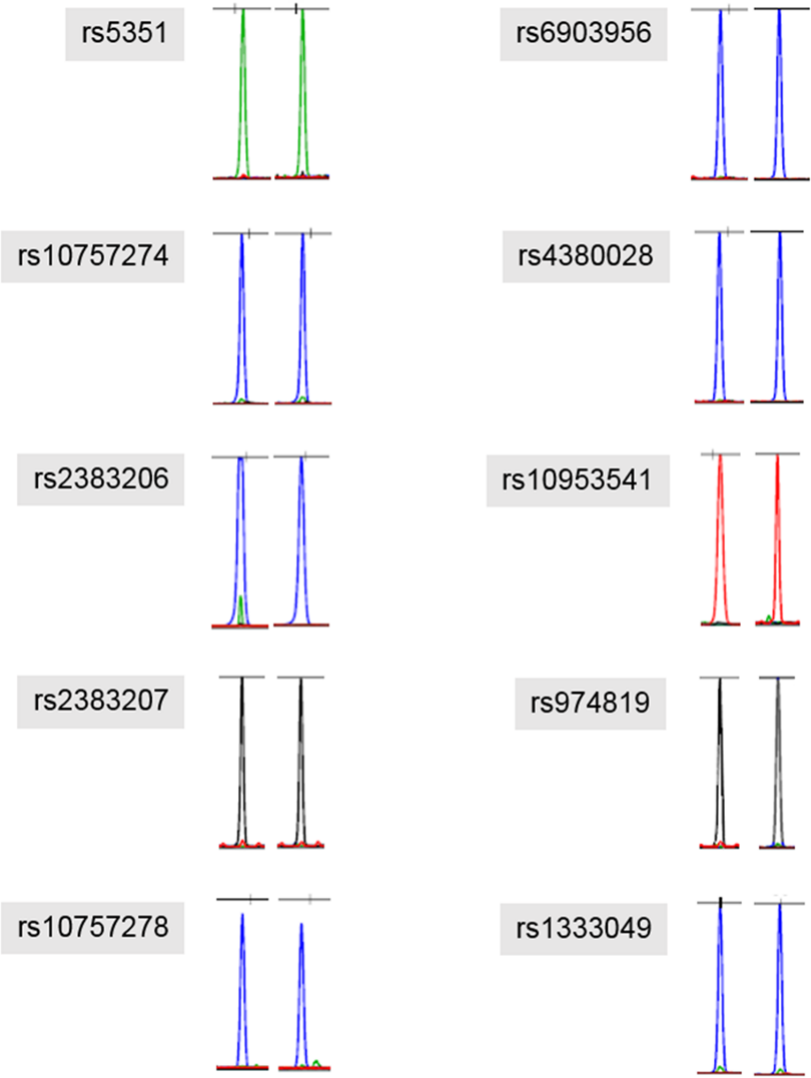
The genotyping results analyzed by the SNaPshot kit were identical to those of Sanger sequencing. Some of the results were shown as antisense allele because SBE primer was made in a reverse direction.

To determine the reliability of our Sanger sequencing results, we re-confirmed them by SNaPshot analysis, this time at a different laboratory. In the results, most of the SNP genotypes thus obtained were identical to those obtained by the earlier Sanger sequencing results. Additionally, rs10757274 was confirmed to be homozygous genotype (GG) (Table 2, Fig 3).

**Fig 4:**
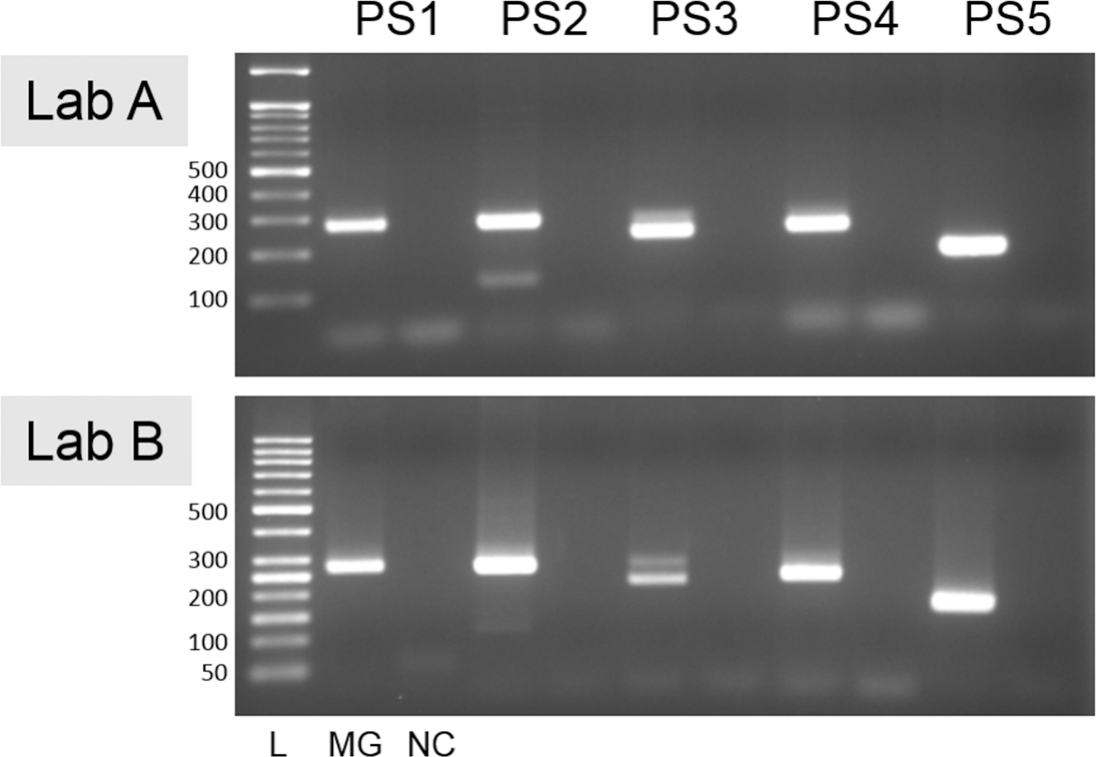
PCR amplification for mtDNA hypervariable region of *Mungyeong* mummy. Electrophoresis of Lab A and B showed the same amplified bands (PS1, 267 bp; PS2, 267 bp; PS3, 226 bp; PS4, 235bp; PS5, 167bp). L, 50bp ladder; MG, *Mungyeong* mummy; NC, negative extraction control.

To rule out any possibility of sample contamination by modern DNA, we compared the haplotypes of the mtDNA control region as obtained from the Joseon mummy and the researchers’ samples. As no identical sequences were found between them, it is highly likely that the obtained data were endogenous (Table 3, Fig 4, S2 File).

**Table 3:** Comparison of mtDNA hypervariable region from *Mungyeong* mummy and researcher samples.

Subjects		Hypervariable region		
		HVI (15991–16390)	HVII (034–369)	HVIII (423–548)
*Mungyeong* mummy	Lab A^a^	16223T 16362C	73G 263G 315.1C	489C
	Lab B^b^	16223T 16362C	73G 263G 315.1C	489C
Researchers	1	16172C 16174T 16223T 16362C	73G 263G 309.1C 315.1C	
	2	16183C 16189C 16220C 16254G 16298C 16362C	73G 249d 263G 315.1C	
	3	16129A 16182C 16183C 16189C 16232A 16249C 16304C 16311C 16344T	73G 152C 249d 263G 315.1C	

^a^cloning

^b^direct sequencing

## Discussion

Atherosclerosis is a pathological state characterized by the thickening and protrusion of intima into the vascular lumen. Although atherosclerosis lesions are benign fatty streaks that do not initially interfere with blood flow, they progress to atheromatous plaque, narrowing the vascular lumen, and eventually inducing fatal diseases such as CAD and its acute complication, MI [50,51]. This is generally called ASCVD, the leading cause of morbidity and mortality in many countries [52].

It is common to think that ASCVD is of concern only for modern people with their various risk factors including hypertension, diabetes, smoking, high caloric diet, and hyperlipidemia, among others [52,53]. However, genetic factors must not be ignored either, as many CAD and atherosclerosis cases have been correlated with the genetic predisposition of the patient [35,54,55]. Thus, in any attempts to determine the health statuses of ancient individuals based on mummified remains, we must not rule out ASCVD as a candidate cause of death either.

In this sense, recent studies on several-hundred-to-thousand-year-old mummies are significant to concerned researchers. Through examinations of actual mummy cases, ASCVD has in fact been common to many individuals in the past. For instance, based on mummy studies, Thompson et al. [56] established that certain ancient individuals suffered from atherosclerosis while alive even though they would have been significantly less exposed to risk factors than we are today. By multiple vascular calcifications on radiological images, atherosclerosis and arteriosclerosis also has been proven a common disease among the ancient Egyptians [57,58]. Pathological signs of atherosclerosis were found in an 18th century Aleutian female mummy [59] and an Italian Renaissance mummy [60] as well. All of this is not even to mention our previous report on 17^th^ century *Mungyeong* mummy that also showed definitive signs of atherosclerosis and CAD [18]. Taking all of this evidence together, we know that ASCVD was a disease not infrequently seen in the ancient world.

Although pioneering studies have contributed greatly to the proper understanding of ASCVD in history, we must also consider the technical limitations of the anatomical and radiological modalities employed. This is in fact a serious issue, given that the samples examined typically are too old for confirmation by ordinary biological-investigative means. Therefore, ancient cases such as the present *Mungyeong* mummy, though showing multiple signs of atherosclerosis and CAD on CT or autopsy, still require novel diagnostics for confirmation.

In this regard, the recent genomic “Iceman” study is very significant to us. In their examination of this 5,300-year-old chalcolithic mummy, Keller et al. [33] found multiple vascular calcifications suggestive of atherosclerosis on CT images. Not satisfied with this evidence, they performed an aDNA analysis in which they also discovered the risk alleles of the SNPs linked to atherosclerosis and CAD. Their detailed sequencing identified the homozygous risk alleles of SNPs rs10757274 and rs2383206, both strong predictors of CAD [33,34]. Their report represents the first successful demonstration of the utility of genetic studies to the diagnosis of ancient atherosclerosis.

The Iceman study, however, is the sole report of its kind, which fact leaves the paleogenetic study of ancient CAD on an unstable basis. Especially for East Asian individuals or populations, which are genetically distant from the Iceman, there is absolutely no report to date on any paleogenetic analysis of ancient ASCVD. Of course, as greater numbers of genetic (aDNA) diagnoses of ancient ASCVD for chronologically and geographically diverse samples become available, the more accurate the information on ASCVD in history will be [34].

In the current study, we thus analyzed ASCVD-related SNPs to determine if any genetic predisposition could be detected in our 17^th^ century Joseon mummy. Among 10 different ASCVD-related SNPs, we eventually found risk alleles in seven SNPs (rs10757274, rs2383206, rs5351, rs2383207, rs10757278, rs4380028, and rs1333049) in Sanger sequencing. These results were verified by SNaPshot analysis. Meanwhile, no risk alleles were identified in SNPs rs6903956, rs10953541 or rs974819.

As noted above, the SNPs examined in this study had already been revealed to be the major risk loci for ASCVD in modern East Asian populations. Four of these (rs10757274, rs2383206, rs2383207, and rs10757278), in chromosome region 9p21, are known to be strongly associated with CAD or MI in various human populations worldwide [36,61–68]. Briefly, the allele GG of rs10757274 has been repeatedly confirmed as a major risk locus for CAD and MI in both Chinese (odd ratio (OR) = 1.37, confidence interval (CI) = 1.31–1.43, p = 7.56E-45) and Korean (OR = 1.29, CI = 1.06–1.58, p = 0.010) populations [37,69]. The association of rs2383206 with CAD and MI phenotypes has been reported based on Chinese (OR = 1.54, CI = 1.18–2.01, p = 0.001) and Korean studies (OR = 1.30, CI = 1.06–1.58, p = 0.024) [37, 65]. The genotype and allelic frequencies for the CAD-related SNP rs2383207 differed remarkably between case and control subjects of Han Chinese (OR = 1.52, CI = 1.13–2.04) and Korean (OR = 1.32, CI = 1.06–1.63, p = 0.001) populations [37, 42]. Researchers also have discovered the contribution of SNP rs10757278 to CAD and acute coronary syndrome (ACS) risks in Han Chinese (OR = 1.91, CI = 1.35–2.68, p = 0.00035) and Korean (OR = 1.29, CI = 1.06–1.57, p = 0.001) populations [37, 70]. In our study, the homozygous risk alleles of the above-noted four different SNPs in chromosome region 9p21 were identified in the *Mungyeong* mummy.

Besides the SNPs on chromosome 9p21, endothelin receptor type B-rs5351 also is known to increase the risk of atherosclerosis in Japanese males (OR of the allele AA-GG = 5.0; CI = 1.13–2.04; p = 0.0187) [35]. The relationship between rs4380028 and CAD has been reported based on a Chinese population study as well (OR = 1.17, p = 0.000667) [69]. The strong associations of rs1333049 with CAD, moreover, have been observed in both Koreans (OR = 1.19, CI = 1.02–1.38, p = 0.025) and Japanese (OR = 1.30, CI = 1.13–1.49, p = 0.00027) [36]. The *Mungyeong* mummy examined in the present study also was homozygous for the risk alleles of the above-noted seven SNPs confirmed to be the major risk loci for atherosclerosis, CAD, and/or MI in East Asian populations. Therefore, we could not deny the possibility that the 17^th^ century Korean mummy had a strong genetic predisposition to increased risk of ASCVD.

## Conclusions

Although radiology and autopsy are still the most useful and convenient techniques for mummy studies, they have drawbacks in terms of confirmative diagnosis of ancient diseases such as ASCVD. Indeed, ancient people must have been less exposed to the relevant risk factors than are people these days. Notwithstanding, the genetic impact on ASCVD onset cannot be ignored either. Thus, research that considers both phenotypes and genotypes is necessary in order to confirm diagnoses of ancient ASCVD.

In this study, to overcome those limitations, we attempted to determine whether paleogenetic analysis can be a useful tool supplementary to the classical ASCVD detection techniques in studies on ancient human remains. By SNP analyses of Sanger sequencing and SNaPshot techniques, we found a genetic predisposition to ASCVD in the Joseon female mummy. Our attempt to genetically diagnose the presence of ASCVD, the first report of its kind outside Europe, is a small but significant step in the effort to make paleopathological disease research more efficient and authentic.

## Supporting information

S1 File Consensus sequence of the ten target loci. (PDF)

S2 File (A) Consensus sequence of mtDNA control region for *Mungyeong* mummy. (B) Comparison between Revised Cambridge Reference Sequence (rCRS, GeneBank accession no. NC_012920), consensus sequence and direct sequencing result of mtDNA control region.
(PDF)
