# Supplementary material for: Paleogenetic study on the 17th century Korean mummy with atherosclerotic cardiovascular disease
Source: PLoS One. 2017 Aug 16;12(8):e0183098. doi: 10.1371/journal.pone.0183098 (PMC5559090; doi:10.1371/journal.pone.0183098)
Supplement: S1 File — (PDF) [file pone.0183098.s001.pdf]

```
rs5351
                      1                            29
Consensus             AAAAGATTGGTGGCTATTCAGTTTCTATT
MgB-01                .............................
MgB-02                .............................
MgB-03                .............................
MgB-04                .............................
MgB-05                .............................
MgB-06                .............................
MgB-07                .............................
MgB-08                .............................
MgB-09                .............................
MgB-10                .............................


rs10757274
                      1                                 34
Consensus             CTGAGTGTTGGGACATAATTGAAATTCACTAGAT
MgB-01                ..................................
MgB-02                ..........A.......................
MgB-03                ..................................
MgB-04                ..................................
MgB-05                ..................................
MgB-06                ..................................
MgB-07                ..................................
MgB-08                ..................................
MgB-09                ..................................
MgB-10                ..................................


rs2383206
                      1                             30
Consensus             TTTTCCTTAGAAATGTTATTGTAGTGTTTG
MgB-01                ..............................
MgB-02                ..............................
MgB-03                ..............................
MgB-04                ..............................
MgB-05                ..............................
MgB-06                ..............................
MgB-07                ..............................
MgB-08                ..............................
MgB-09                ..............................


rs2383207
                      1                                                                                81
Consensus             ATTTTTTACTCCTGTTCGGATCCCTTCGGCTAAGCATGATTATTTACTATTTTCAGCTATTAGTTATGTCTTGTTGAAAAA
MgB-01                .................................................................................
MgB-02                .................................................................................
MgB-03                .................................................................................
MgB-04                .................................................................................
MgB-05                .................................................................................
MgB-06                .................................................................................
MgB-07                .................................................................................
MgB-08                .................................................................................
MgB-09                .................................................................................
MgB-10                .................................................................................


rs10757278
                      1                                      39
Consensus             AGGGTGTGGTCATTCCGGTAGGCAGCGATGCAGAATCAA
MgB-01                .......................................
MgB-02                .......................................
MgB-03                .......................................
MgB-04                .......................................
MgB-05                .......................................
MgB-06                .......................................
MgB-07                .......................................
MgB-08                .......................................
MgB-09                .......................................
MgB-10                ..........................A............
```

rs6903956

```
           1                                                              64
Consensus  TCAAAAATAAATAAATAAATAAATAAATAAATAGTGCCATAGGTTATTACTTAAGGTTGGTCCC
MgB-01     ................................................................
MgB-02     ................................................................
MgB-03     ................................................................
MgB-04     ................................................................
MgB-05     ................................................................
MgB-06     ................................................................
MgB-07     ................................................................
MgB-08     ................................................................
```

rs4380028

```
           1                                                                          76
Consensus  AGTAACTTGCCCAACGTCCCACATTTGGAAGTGTCGGGGCCAGGACTGGCCTAATTCCAGTGCCTGGATTTCTAAT
MgB-01     ............................................................................
MgB-02     ............................................................................
MgB-03     ............................................................................
MgB-04     ............................................................................
MgB-05     ............................................................................
MgB-06     ............................................................................
MgB-07     ............................................................................
MgB-08     ............................................................................
MgB-09     ............................................................................
```

rs10953541

```
           1                                                                                   85
Consensus  AAACTTTAAATAAATTTCTTAAAGCATATACTAACAATCAGAAACTGAGTGTAAAGACCTATTATGGGTACCTAAGTATTAGCAG
MgB-01     .....................................................................................
MgB-02     .....................................................................................
MgB-03     .....................................................................................
MgB-04     .....................................................................................
MgB-05     .....................................................................................
MgB-06     .....................................................................................
MgB-07     .....................................................................................
MgB-08     .....................................................................................
MgB-09     .....................................................................................

           86                   108
Consensus  CATGCAATAGAGATACTAGATTT
MgB-01     .......................
MgB-02     .......................
MgB-03     .......................
MgB-04     ............G..........
MgB-05     .......................
MgB-06     .......................
MgB-07     .......................
MgB-08     .......................
MgB-09     .......................
```

rs974819

```
           1                                                                                   85
Consensus  TTTAAGGAAATATATTTTTCTGTTTCTACTGTTTTATTTTCATGTTTGGAGAAGCAGACTATTATTCTCATTTTCTTTTGTTTCA
MgB-01     .....................................................................................
MgB-02     .....................................................................................
MgB-03     .....................................................................................
MgB-04     .....................................................................................
MgB-05     .....................................................................................
MgB-06     .....................................................................................
MgB-07     .....................................................................................
MgB-08     .....................................................................................
MgB-09     .....................................................................................
MgB-10     .....................................................................................

           86                             118
Consensus  TAAAAAAAATGAAAAACCCAGCTTATATAATGT
MgB-01     .................................
MgB-02     .................................
MgB-03     .................................
MgB-04     .................................
MgB-05     .................................
MgB-06     .................................
MgB-07     .................................
MgB-08     .................................
MgB-09     .................................
MgB-10     .................................
```

rs1333049

```
                  1                                                                                   85
Consensus         ATGACACTTCTTAGGCTATCATTTCATTCCAAATTTATGGTCACTACCCTACTGTCATTCCTCATACTAATCATATGATCAACAG
MgB-01            .....................................................................................
MgB-02            .....................................................................................
MgB-03            .....................................................................................
MgB-04            .....................................................................................
MgB-05            .............................................................................G.......
MgB-06            .....................................................................................
MgB-07            .....................................................................................
MgB-08            .....................................................................................

                  86         97
Consensus         TTCAAAAGCAGC
MgB-01            ............
MgB-02            ............
MgB-03            ............
MgB-04            ............
MgB-05            ............
MgB-06            ............
MgB-07            ............
MgB-08            ............
```
