# Supplementary material for: Paleogenetic study on the 17th century Korean mummy with atherosclerotic cardiovascular disease
Source: PLoS One. 2017 Aug 16;12(8):e0183098. doi: 10.1371/journal.pone.0183098 (PMC5559090; doi:10.1371/journal.pone.0183098)
Supplement: S2 File — (A) Consensus sequence of mtDNA control region for Mungyeong mummy. (B) Comparison between Revised Cambridge Reference Sequence (rCRS, GeneBank accession no. NC_012920), consensus sequence and direct sequencing result of mtDNA control region. (PDF) [file pone.0183098.s002.pdf]

(A) Consensus sequence of mtDNA control region for Mungyeong mummy

PS1

```
              1                                                                                                                   120
Consensus     CAAAGCTAAGATTCTAATTTAAACTATTCTCTGTTCTTTCATGGGGAAGCAGATTTGGGTACCACCCAAGTATTGACTCACCCATCAACAACCGCTATGTATTTCGTACATTACTGCCAG
MgB-PS1-01    ........................................................................................................................
MgB-PS1-02    ........................................................................................................................
MgB-PS1-03    ........................................................................................................................
MgB-PS1-04    ........................................................................................................................
MgB-PS1-05    ........................................................................................................................
MgB-PS1-06    ........................................................................................................................
MgB-PS1-07    ........................................................................................................................
MgB-PS1-08    ........................................................................................................................
MgB-PS1-09    ........................................................................................................................
MgB-PS1-10    ........................................................................................................................
MgB-PS1-11    ..............G.........................................................................................................

              121                                                                                                     227
Consensus     CCACCATGAATATTGTACGGTACCATAAATACTTGACCACCTGTAGTACATAAAAACCCAATCCACATCAAAACCCCCTCCCCATGCTTACAAGCAAGTACAGCAAT
MgB-PS1-01    ...........................................................................................................
MgB-PS1-02    ...........................................................................................................
MgB-PS1-03    ...........................................................................................................
MgB-PS1-04    ...........................................................................................................
MgB-PS1-05    ...........................................................................................................
MgB-PS1-06    ...........................................................................................................
MgB-PS1-07    ...........................................................................................................
MgB-PS1-08    ...........................................................................................................
MgB-PS1-09    ...........................................................................................................
MgB-PS1-10    ...........................................................................................................
MgB-PS1-11    ...........................................................................................................
```

PS2

```
              1                                                                                                                   120
Consensus     AAACCCAATCCACATCAAAACCCCCTCCCCATGCTTACAAGCAAGTACAGCAATCAACCTTCAACTATCACACATCAACTGCAACTCCAAAGCCACCCCTCACCCACTAGGATACCAACA
MgB-PS2-01    ........................................................................................................................
MgB-PS2-02    ........................................................................................................................
MgB-PS2-03    ................................................................................................................C.......
MgB-PS2-04    ........................................................................................................................
MgB-PS2-05    ........................................................................................................................
MgB-PS2-06    ...................................................................C....................................................
MgB-PS2-07    ........................................................................................................................
MgB-PS2-08    ........................................................................................................................
MgB-PS2-09    ........................................................................................................................
MgB-PS2-10    ........................................................................................................................
MgB-PS2-11    ........................................................................................................................

              121                                                                                                     227
Consensus     AACCTACCCACCCTTAACAGTACATAGTACATAAAGCCATTTACCGTACATAGCACATTACAGTCAAATCCCTTCTCGCCCCCATGGATGACCCCCCTCAGATAGGG
MgB-PS2-01    ...........................................................................................................
MgB-PS2-02    ...........................................................................................................
MgB-PS2-03    ...........................................................................................................
MgB-PS2-04    ...........................................................................................................
MgB-PS2-05    ...........................................................................................................
MgB-PS2-06    ...........................................................................................................
MgB-PS2-07    ...........................................................................................................
MgB-PS2-08    ...........................................................................................................
MgB-PS2-09    ...........................................................................................................
MgB-PS2-10    .....................................................................................................C.....
MgB-PS2-11    ...........................................................................................................
```

PS3

```
                1                                                                                                                     120
Consensus       GGAGCTCTCCATGCATTTGGTATTTTCGTCTGGGGGGTGTGCACGCGATAGCATTGCGAGACGCTGGAGCCGGAGCACCCTATGTCGCAGTATCTGTCTTTGATTCCTGCCTCATCCTAT
MgB-PS3-01      ........................................................................................................................
MgB-PS3-02      ........................................................................................................................
MgB-PS3-03      ........................................................................................................................
MgB-PS3-04      ........................................................................................................................
MgB-PS3-05      ........................................................................................................................
MgB-PS3-06      ........................................................................................................................
MgB-PS3-07      ........................................................................................................................
MgB-PS3-08      ........................................................................................................................
MgB-PS3-09      ........................................................................................................................
MgB-PS3-10      ........................................................................................................................

                121                                                            185
Consensus       TATTTATCGCACCTACGTTCAATATTACAGGCGAACATACTTACTAAAGTGTGTTAATTAATTAA
MgB-PS3-01      .................................................................
MgB-PS3-02      .................................................................
MgB-PS3-03      .................................................................
MgB-PS3-04      .................................................................
MgB-PS3-05      .................................................................
MgB-PS3-06      .................................................................
MgB-PS3-07      .................................................................
MgB-PS3-08      .................................................................
MgB-PS3-09      .................................................................
MgB-PS3-10      .................................................................
```

PS4

```
                1                                                                                                                     120
Consensus       AATATTACAGGCGAACATACTTACTAAAGTGTGTTAATTAATTAATGCTTGTAGGACATAATAATAACAATTGAATGTCTGCACAGCCGCTTTCCACACAGACATCATAACAAAAAATTT
MgB-PS4-01      ........................................................................................................................
MgB-PS4-02      ........................................................................................................................
MgB-PS4-03      ........................................................................................................................
MgB-PS4-04      ....................C...................................................................................................
MgB-PS4-05      .........................G..............................................................................................
MgB-PS4-06      ........................................................................................................................
MgB-PS4-07      ........................................................................................................................
MgB-PS4-08      ........................................................................................................................

                121                                                                       196
Consensus       CCACCAAAccccccctccccccGCTTCTGGCCACAGCACTTAAACACATCTCTGCCAAACCCCAAAAACAAAGAAC
MgB-PS4-01      ............................................................................
MgB-PS4-02      ............................................................................
MgB-PS4-03      ............................................................................
MgB-PS4-04      ............................................................................
MgB-PS4-05      ............................................................................
MgB-PS4-06      ............................................................................
MgB-PS4-07      ............................................................................
MgB-PS4-08      ............................................................................
```

PS5

```
                1                                                                                                                          125
Consensus       TAACAGTCACCCCCCAACTAACACATTATTTTCCCCTCCCACTCCCATACTACTAATCTCATCAACACAACCCCCGCCCATCCTACCCAGCACACACACACCGCTGCTAACCCCATACCCCGAAC
MgB-PS5-01      .............................................................................................................................
MgB-PS5-02      .............................................................................................................................
MgB-PS5-03      .............................................................................................................................
MgB-PS5-04      .............................................................................................................................
MgB-PS5-05      .............................................................................................................................
MgB-PS5-06      .............................................................................................................................
MgB-PS5-07      .............................................................................................................................
MgB-PS5-08      .............................................................................................................................
MgB-PS5-09      .............................................................................................................................
```

(B) Comparison between rCRS, consensus sequence and direct sequencing result of mtDNA control region

Hypervariable region 1

```
                    15991                                                                                                          16110
rCRS                CAAAGCTAAGATTCTAATTTAAACTATTCTCTGTTCTTTCATGGGGAAGCAGATTTGGGTACCACCCAAGTATTGACTCACCCATCAACAACCGCTATGTATTTCGTACATTACTGCCAG
Consensus           ........................................................................................................................
Direct (PS1-FWD)                         ...................................................................................................
Direct (PS1-REV)    ........................................................................................................................

                    16111                                                                                                          16230
rCRS                CCACCATGAATATTGTACGGTACCATAAATACTTGACCACCTGTAGTACATAAAAACCCAATCCACATCAAAACCCCCTCCCCATGCTTACAAGCAAGTACAGCAATCAACCCTCAACTA
Consensus           ................................................................................................................T.......
Direct (PS1-FWD)    ........................................................................................
Direct (PS1-REV)    ...........................................................................................................
Direct (PS2-FWD)                                                                           .........................................T.......

                    16231                                                                                                          16350
rCRS                TCACACATCAACTGCAACTCCAAAGCCACCCCTCACCCACTAGGATACCAACAAACCTACCCACCCTTAACAGTACATAGTACATAAAGCCATTTACCGTACATAGCACATTACAGTCAA
Consensus           ........................................................................................................................
Direct (PS2-FWD)    ........................................................................................................................
Direct (PS2-REV)                        ....................................................................................................

                    16351                                  16390
rCRS                ATCCCTTCTCGTCCCCATGGATGACCCCCCTCAGATAGGG
Consensus           ...........C............................
Direct (PS2-FWD)    ...........C............................
Direct (PS2-REV)    ...........C..........
```

Hypervariable region 2

```
                    35                                                                                                               154
rCRS                GGAGCTCTCCATGCATTTGGTATTTTCGTCTGGGGGGTATGCACGCGATAGCATTGCGAGACGCTGGAGCCGGAGCACCCTATGTCGCAGTATCTGTCTTTGATTCCTGCCTCATCCTAT
Consensus           ......................................G.................................................................................
Direct (PS3-FWD)                    ......................G.................................................................................
Direct (PS3-REV)    ......................................G.................................................................................

                    155                                                                                                              274
rCRS                TATTTATCGCACCTACGTTCAATATTACAGGCGAACATACTTACTAAAGTGTGTTAATTAATTAATGCTTGTAGGACATAATAATAACAATTGAATGTCTGCACAGCCACTTTCCACACA
Consensus           ............................................................................................................G...........
Direct (PS3-FWD)    .................................................................
Direct (PS3-REV)    .................................................
Direct (PS4-FWD)                                                  ..............................................................G...........

                    275                                                                                         369
rCRS                GACATCATAACAAAAAATTTCCACCAAAccccccctccccc-GCTTCTGGCCACAGCACTTAAACACATCTCTGCCAAACCCCAAAAACAAAGAAC
Consensus           .........................................C......................................................
Direct (PS4-FWD)    .........................................C......................................................
Direct (PS4-REV)                                .............C....................................
```

Hypervariable region 3

```
                    424                                                                                                                   548
rCRS                TAACAGTCACCCCCCAACTAACACATTATTTTCCCCTCCCACTCCCATACTACTAATCTCATCAATACAACCCCCGCCCATCCTACCCAGcacacacacaccgctgctaaccccataccccgaac
Consensus           .................................................................C...........................................................
Direct (PS5-FWD)                       ..............................................C...........................................................
```
